# Effects of isoflurane, ketamine-xylazine and a combination of medetomidine, midazolam and fentanyl on physiological variables continuously measured by telemetry in Wistar rats

**DOI:** 10.1186/s12917-014-0198-3

**Published:** 2014-08-23

**Authors:** Maike Albrecht, Julia Henke, Sabine Tacke, Michael Markert, Brian Guth

**Affiliations:** 1 Department of Nonclinical Drug Safety, Biological Laboratory Service, Boehringer Ingelheim Pharma GmbH & Co. KG, Birkendorfer Str. 65, Biberach, 88397, Germany; 2 Department of Veterinary Clinical Sciences, Clinic for Small Animals-Surgery, Justus-Liebig University, Frankfurter Str. 108, Giessen, 35392, Germany; 3 Department of Drug Discovery Support, General Pharmacology, Boehringer Ingelheim Pharma GmbH & Co. KG, Birkendorfer Str. 65, Biberach, 88397, Germany

**Keywords:** Rat, Anaesthesia, Isoflurane, Ketamine-xylazine, Medetomidine-midazolam-fentanyl, Telemetry, Heart rate, Blood pressure, Core body temperature

## Abstract

**Background:**

This study investigated effects on cardiovascular parameters during anaesthesia with isoflurane (ISO, 2–3 Vol%), ketamine-xylazine (KX, 100 mg•kg^−1^ + 5 mg•kg^−1^) or a combination of medetomidine-midazolam-fentanyl (MMF, 0.15 mg•kg^−1^ + 2.0 mg•kg^−1^ + 0.005 mg•kg^−1^) in rats throughout induction, maintenance and recovery from anaesthesia. Rats were instrumented with a telemetric system for the measurement of systolic, diastolic and mean arterial pressure (SAP, DAP, MAP), pulse pressure (PP), heart rate (HR) and core body temperature (BT). The parameters were continuously measured before, during and after each type of anaesthesia. Forty minutes after induction, ISO delivery was terminated and MMF was antagonized with atipamezole-flumazenil-naloxone (AFN, 0.75 mg•kg^−1^ + 0.2 mg•kg^−1^ + 0.12 mg•kg^−1^) whereas KX was not antagonized.

**Results:**

Differences were observed between anaesthesias with KX (301 min) lasting much longer than MMF (45 min) and ISO (43 min). HR in ISO ($\bar{x}$ = 404 ± 25 bpm) increased during the time of surgical tolerance whereas a HR decrease was observed in KX ($\bar{x}$ = 255 ± 26 bpm) and MMF ($\bar{x}$ = 209 ± 24 bpm). In ISO (MAP during time of surgical tolerance: $\bar{x}$ = 89 ± 12.3 mmHg) and KX (MAP during wake-up period: $\bar{x}$ = 84 ± 8.5 mmHg) mild hypotensive values were observed, whereas blood pressure (BP) in MMF (MAP during time of surgical tolerance: $\bar{x}$ = 138 ± 9.9 mmHg) increased. Despite keeping animals on a warming pad, a loss of BT of about 1°C was seen in all groups. Additionally, we observed a peaked increase of HR ($\bar{x}$ = 445 ± 20 bpm) during the wake-up period with ISO and an increase of PP ($\bar{x}$ = 59 ± 8.5 mmHg) in MMF during the time of surgical tolerance.

**Conclusion:**

The anaesthesias influenced very differently the cardiovascular parameters measured in Wistar rats. ISO caused mild hypotension and increased HR whereas MMF produced a marked hypertension and a significant decrease of HR. The slightest alterations of BP, HR and BT were observed using KX, but the long wake-up and recovery period suggest the need for prolonged monitoring.

## Background

The rat is a commonly used animal in research and for many experimental procedures anaesthesia is required or recommended [1],[2]. In the selection of an optimal anaesthetic agent to use, the considerations include: 1) ease of handling, 2) a low-stress induction and recovery, 3) minimal impact on physiological parameters and thereby the research results, 4) the ability to adjust the level of anaesthesia or to prolong it if necessary and, when needed, 5) a rapid recovery from the anaesthesia. Since a given anaesthesia regime may influence experimental outcomes, the choice of a suitable anaesthesia has to be considered carefully in regard to the experimental requirements [1],[3]. The influence of different anaesthetics on cardiovascular and respiratory parameters, body temperature, circadian rhythm, blood values, locomotor activity, intracranial pressure or even on cognitive skills has been the subject of previous studies [4]–[14]. It was also shown that responses to anaesthetics may be strain-dependent [1],[15]. However, the methods for assessing effects on arterial blood pressure (BP) in many studies were limited, data collection was too short, or the data analysis was inadequate to detect potentially important alterations. Thus, the purpose of this study was to provide an accurate and continuous assessment of the cardiovascular effects of three commonly used anaesthetic regimen in rats. We therefore compared inhalational anaesthesia using isoflurane (ISO), with an intramuscular (i.m.) combination of ketamine and xylazine (KX) as well as a completely antagonizable anaesthesia using the combination of medetomidine, midazolam and fentanyl (MMF) administered i.m. in adult male Wistar rats instrumented for the continuous, telemetric collection of cardiovascular parameters.

The use of ISO increased over the last years, although specialized equipment is required for an inhalational anaesthesia [16]. ISO has little influence on metabolism because of an almost complete elimination via exhaled air and it offers simple handling in deepening and prolongation of anaesthesia which could be reasons for its increased use [1],[3],[17]. However, for painful procedures one should supplement ISO with analgesics due to its weak analgesic properties [18]. KX is a routinely used anaesthetic mixture in laboratory animals, although it is not always recommended as a suitable anaesthetic combination for use specifically in rats [3],[16],[19]–[21]. MMF is commonly used in Germany, although it is not as common as KX or ISO in other countries. Therefore, experimental data concerning MMF anaesthesia are limited. This anaesthesia offers the advantage of a rapid and complete reversal by using an injection of atipamezole, flumazenil and naloxone (AFN) [20],[22]–[25]. Anaesthesia in rodents influences diurnal rhythm and often results in hypothermia and hypoglycemia because of their high metabolic rate and their large surface area to body weight ratio [3],[20],[26]–[28]. Due to a fast resumption of food and water intake because of a very short recovery period when antagonized and, by our experience, its excellent survival rate, MMF seems to be advantageous over other anaesthesias in rodents. One important point to consider with MMF is the reversal of analgesia by naloxone. Postoperative pain management has to be provided by other compounds than opioids or one has to keep into consideration a partial antagonization with an opioid-agonist/antagonist or partial agonist such as butorphanol or buprenorphine [29]–[32]. Despite these possible advantages or disadvantages of these three anaesthesia regimes, there is little data on their cardiovascular effects throughout induction, maintenance and recovery from the anaesthesia, effects that could be decisive for the selection of the optimal anaesthesia for a given setting.

To ensure high quality data, we used rats implanted with a telemetry device to provide continuous, state-of-the-art monitoring of cardiovascular parameters [33]–[36]. Telemetry technology allows group-housing conditions and measurement in freely moving rats, without the need for a stressful restraint. The data is also not influenced by an additional anaesthesia performed a few days or only hours prior to an experiment, to implant an exteriorized catheter for measuring blood pressure [37].

The aim of this study was to show alterations in cardiovascular parameters including systolic arterial pressure (SAP), diastolic arterial pressure (DAP), mean arterial pressure (MAP), pulse pressure (PP), heart rate (HR) and core body temperature (BT) during ISO, KX and MMF anaesthesia in male Wistar rats at specific stages of anaesthesia (induction, maintenance and recovery).

## Methods

### Animals

Twelve, male Wistar rats with a mean body weight of 287 ± 30 g were acquired from a commercial breeder (Charles River Laboratories, Sulzfeld, Germany). These animals were housed in groups of three in a Makrolon® cage (Type IV) containing a wooden bedding material (Lignocel select fine, J. Rettenmaier & Söhne GmbH + Co. KG, Rosenberg, Germany). Cage bedding changes were performed twice weekly. Two red, transparent plastic tubes, nesting material and a wooden chewing block were provided in each cage for animal enrichment. The rats received a commercially available diet (3438 maintenance diet, KLIBA NAFAG, Provimi Kliba AG, Kaiseraugst, Switzerland) and tap water *ad libitum*. The animal room was maintained at 22 ± 2°C and 55 ± 10% relative humidity and there was an air change of at least 15 cycles/hour. Light was on from 6:00 am to 6:00 pm, starting and ending with a dimmer-period of 30 minutes. Together with the light, a radio was turned on. The rats were allowed to acclimate to the housing conditions and the husbandry procedures for at least two weeks prior to the surgical implantation of the radiotelemetry transmitter (see below).

### Implantation of the radiotelemetry transmitter

The implantation of the transmitter (DSI PhysioTel™ C50-PXT) was performed under general anaesthesia using MMF in the same dosage as described below in this study. One third of the initial dosage was administered again after 45 minutes in order to maintain anaesthesia. For analgesia, the rats received prior to surgery 50 mg•kg^−1^ metamizole i.m. (Novalgin®, 500 mg•ml^−1^, Sanofi Aventis, Frankfurt/Main, Germany) and 1 mg•kg^−1^ meloxicam s.c. (Metacam®, 20 mg•ml^−1^, Boehringer Ingelheim, Ingelheim/Rhein, Germany). Metamizole application was repeated three times until the next day. To prevent a bacterial infection, 10 mg•kg^−1^ enrofloxacin (Baytril® 2.5% ad us. vet., Bayer, Leverkusen, Germany) was administered subcutaneously after the analgesic injections. As soon as the rats lost their righting reflex, the ventral site of the rat was shaved and then disinfected with Kodan®-spray (Schülke & Mayr, Norderstedt, Germany) and Betaisodona®-solution (Mundipharma GmbH, Limburg (Lahn), Germany). Protective eye lubricant (VitA-POS, Ursapharm, Saarbrücken, Germany) was administered to both eyes, loss of body temperature was minimized using a warm water heating pad and supplemental oxygen was provided through a nose cone. Once a surgical level of anaesthesia was confirmed through the loss of reflexes, an incision was made in the *linea alba* from below the sternum to the umbilical region to open the abdominal cavity. The intestinal tract was carefully repositioned using a moist swab in a cranial direction to exposure the *aorta abdominalis* which was dissected free for insertion of the blood pressure catheter of the telemetry unit. Blood flow was temporarily stopped using two vascular clips and the catheter was inserted between them and fixed in place with tissue glue (Histoacryl®, B.Braun, Aesculap AG, Tuttlingen, Germany). Clips and the swab were then removed and the transmitter was sutured to the abdominal wall (Mersilene® 3–0, Ethicon®, Johnson-Johnson Medical GmbH, Norderstedt, Germany). The ECG leads were exteriorized through the abdominal muscle layer and placed subcutaneously, one to the end of the sternum and the other to the ventral region of the trachea. The ECG leads were sutured to the nearby muscle tissue (Mersilene® 3–0, Ethicon®, Johnson-Johnson Medical GmbH, Norderstedt, Germany). The abdominal cavity was closed in layers with first a muscle and then a skin suture (Vicryl® 3–0, Ethicon®, Johnson-Johnson Medical GmbH, Norderstedt, Germany). At the end of the procedure the rats received 20 ml•kg^−1^warmed lactated ringer’s solution (Ringer-Lactat nach Hartmann B. Braun, B. Braun, Melsungen, Germany) subcutaneously. The anaesthesia was then antagonized with a subcutaneous injection of AFN (same dosage as used in this study, see below). The whole implantation procedure took 90 minutes on average. Meloxicam was administered once-daily for two additional days. The rats were allowed two weeks to recover from this surgical procedure before entering the study.

### Experimental design

The three different anaesthetic treatments were evaluated using a randomized, crossover design with each rat receiving each of the following three anaesthetic treatments on different days: 1) an inhalational anaesthesia with isoflurane for 40 minutes (Forene® 100% (V/V), Abbott, Wiesbaden, Germany), 2) a combination of ketamine (Ketavet®, 100 mg•ml^−1^, Pfizer, Berlin, Germany) and xylazine (Rompun® 2%, 20 mg•ml^−1^, Bayer, Leverkusen, Germany) administered intramuscularly and 3) a combination of medetomidine (Domitor, 1 mg•ml^−1^, Orion Pharma, Espoo, Finland), midazolam (Dormicum®, 5 mg/ml, Roche, Grenzach-Wyhlen, Germany) and fentanyl (Fentanyl®-Janssen, 0.05 mg•ml^−1^, Janssen, Wien, Austria) administered intramuscularly and reversed after 40 minutes with a subcutaneous injection of atipamezole (Antisedan®, 5 mg•ml^−1^, Orion Pharma, Espoo, Finland), flumazenil (Flumazenil Hexal®, 0.1 mg•ml^−1^, Hexal, Holzkirchen, Germany) and naloxone (Naloxon Inresa, 0.4 mg•ml^−1^, Inresa, Freiburg, Germany). With KX anaesthesia the rats received no treatment to reverse the anaesthesia. The rats were allowed a recovery period of two weeks between the anaesthesias. To allow for a thorough monitoring of each individual rat during anaesthesia, only three anaesthesias were performed per day. SAP, DAP, MAP, PP, HR and BT were continuously measured from the start of measurements (6.00 am) until the end of measurements (~5 pm). A complete measurement consisted of a 2–4 hour pretreatment acclimatization period for reaching baseline values, the time for performing anaesthesia (a duration of 40 minutes was chosen for ISO and MMF anaesthesia; KX anaesthesia lasted in some animals up to 395 minutes, because it was not reversed), a wake-up and a recovery period. All anaesthesias were performed by the same veterinarian to reduce variability.

The experimental procedures were approved by the Animal Care and Ethics Committee of the Regional Authority in Tuebingen, Baden-Wuerttemberg, Germany (Approval number: 12–038).

### Procedure

Before starting the telemetric measurement, each rat was weighed and placed individually into a Makrolon® cage, containing bedding material and a red, transparent plastic tube and covered with a cloth. Single housing and withdrawal of food were required, because rats had come to rest to assess individual baseline values. Tap water was provided in a water bottle during the entire time of data collection. Each cage was placed on a radiofrequency receiver plate. A water heating pad was placed between the cage and the telemetric receiver plate and it was left on (38°C) throughout the study. Data collection started by switching on the radiotransmitter using a magnet, the operator left the room and the animals were given at least two hours to establish resting, baseline conditions. Afterwards, the induction of anaesthesia was started with the first rat. It was of note that this procedure seemed to have no impact on the cardiovascular parameters measured from the other two rats in the room. Their cages were still covered with a cloth and anaesthesia was performed as quiet as possible so that the produced noise did not drown out the radio, which was switched on at the beginning of measurements. Each rat received protective eye lubricant (VitA-POS, Ursapharm, Saarbrücken, Germany) at the beginning of anaesthesia. After induction of anaesthesia, the righting reflex (defined as positive, when a rat, placed on its back/side, immediately turns over to the normal position with all four feet on the ground) and pedal withdrawal reflex on the hind and forelimbs were monitored and classified (+, ±, −) after 2.5, 5, 7.5, 10, 15, 20, 30, 40, 42.5, 45, 47.5, 50, 60, 70… minutes until the righting reflex had returned. The time until loss and later regaining of the righting reflex was determined. Based on the presence or absence of the reflexes assessed, the anaesthesia time was divided into the following intervals: 1) the **induction time** defined as the time from application of the anaesthetic(s) to loss of the righting reflex, 2) the **time of non-surgical tolerance**, defined as the time from the loss of the righting reflex until loss of the pedal withdrawal reflexes on the hind and forelimbs, 3) the **time of surgical tolerance**, defined as the time from the absence of righting and pedal withdrawal reflexes until regaining at least one pedal withdrawal reflex, 4) **the wake-up period**, describing the time from regaining one pedal withdrawal reflex until regaining the righting reflex and 5) the **time of recovery**, defined as the time from regaining the righting reflex until the end of measurements. The measurements were terminated not earlier than six hours after induction for all three anaesthesias. Because of long-lasting effects of KX, measurements continued for at least two hours after the rats regained the righting reflex.

The different anaesthetic treatments were performed as described below.

### ISO

A whole body chamber was prefilled with 5 Vol % ISO in 100% oxygen. The chamber was placed on the transmitter receiver plate and the rat was positioned into the chamber and the time was measured till loss of the righting reflex. To assess the righting reflex, the chamber with the animal was tipped over. The rat was then placed in dorsal recumbency in the middle of the water heating pad and 5 Vol % ISO was further administered using a nose cone. The concentration of ISO was then individually regulated and reduced to 2–3 Vol % for producing a depth of anaesthesia suitable for surgical procedures, which meant that all reflexes tested had to disappear. Forty minutes after induction of anaesthesia, ISO administration was stopped and the rat was put back in its cage on the receiver. The rat was positioned on its back, so that return of the righting reflex could be determined, when the animal turned around to a ventral recumbency.

### KX

Ketamine (100 mg•kg^−1^) and xylazine (5 mg•kg^−1^) were mixed together in one syringe. The volume of the KX injection was too large (1.25 ml•kg^−1^) to be administered in one hind leg, therefore, it was divided in half and injected intramuscularly in the caudal parts of the femoral musculature of both hind legs. For a continuous telemetric measurement even during this procedure, the injection was performed next to the receiver plate to assure capture of the telemetric signals. After loss of the righting reflex, the rat was placed on its back in the middle of the heating pad and supplied with 100% oxygen using a nose cone. Due to the fact that the duration of this anaesthesia could not be accurately predicted, the rat stayed on the heating pad until its righting reflex returned. Thereafter, the animal was immediately placed back in its cage located on the receiver. The long sleeping and recovery time in KX anaesthesia necessitated the administration of fluids and therefore all rats received 5 ml of warmed lactated ringer’s solution (Ringer-Lactat nach Hartmann B. Braun, B. Braun, Melsungen, Germany) subcutaneously one hour after induction.

### MMF

Medetomidine (0.15 mg•kg^−1^), midazolam (2.0 mg•kg^−1^) and fentanyl (0.005 mg•kg^−1^) were mixed in one syringe (total volume: 0.65 ml•kg^−1^) and were administered intramuscularly in the caudal part of the femoral musculature of one hind leg. After loss of the righting reflex the cage was removed and the rat was placed in dorsal recumbency in the middle of the heating pad and 100% oxygen was provided using a nose cone. The antagonists atipamezole (0.75 mg•kg^−1^), flumazenil (0.2 mg•kg^−1^) and naloxone (0.12 mg•kg^−1^) were mixed in one syringe and administered subcutaneously 40 minutes after induction of the anaesthesia. Thereafter, the rat was returned to its cage in dorsal recumbency to determine the moment of righting.

### Statistical analysis

NOTOCORD-hem™ was used for telemetric data acquisition and data were further evaluated using MS Excel. Values for each parameter over 10 minutes were summarized by the assessment of mean baseline values. Baseline values were calculated as the mean of the measurements starting 60 up to 10 minutes before the induction of anaesthesia. Mean values measured during and after anaesthesia were calculated with medians based on 20 second intervals. For data import from MS Excel the software package SAS 9.2 was used. The statistical evaluation was done using the software package SAS 9.3 (SAS Institute Inc., Cary, North Carolina, USA). The statistical evaluation was done for each parameter measured (SAP, DAP, MAP, PP, HR and BT) and each of the defined intervals (induction time, time of non-surgical tolerance, time of surgical tolerance, wake-up and recovery period). The area under the curve (AUC) was calculated for each animal individually using the trapezoidal rule for the intervals. The different intervals were compared to the baseline interval by an analysis of variance (ANOVA) for repeated measurements for every anaesthesias separately. Effects were quantified by mean differences and their two-sided 95% confidence interval. Additionally, the parameters were analysed by a one-factorial analysis of covariance (ANCOVA) with heteroscedastic variances and the fix factor treatment including the baseline as covariate. The following comparisons were performed by two sided t-tests:

 ISO vs. KX,

 ISO vs. MMF,

 KX vs. MMF.

Treatment effects were quantified by mean differences, based on the adjusted mean values, and their two-sided 95% confidence interval. The level of significance for both analysis was fixed at α = 5%. A p-value less than 0.05 was considered to be statistically significant.

## Results

In the course of experiments three rats developed a poor quality blood pressure signal; in two rats it occurred between the first and the second anaesthesia and in one rat between the second and third anaesthesia. Therefore two ISO, two MMF and one KX measurements were not available for the statistical analysis. Furthermore an anaesthetic emergency occurred during one MMF anaesthesia and one rat receiving KX anaesthesia never reached the anaesthetic stage of surgical tolerance. These animals were excluded from further analysis. Consequently, ten ISO, ten KX and nine MMF measurements were included in the statistical analysis.

All haemodynamic data and body temperature are summarized in Table 1 and the statistical analysis for the comparison of anaesthetic treatments is found in Table 2. There were no relevant differences in baseline values among the three treatment groups. The PP in KX (25 mmHg) was, however, slightly lower than that seen in ISO (31 mmHg) or MMF (31 mmHg).

**Table 1:** Mean values with standard deviation

**Parameter**	**Treatment**	**Baseline**	**Induction time**	**Non-surgical tolerance**	**Surgical tolerance**	**Wake-up period**	**Recovery**
		**Mean ± SD**	**Mean ± SD**	**Mean ± SD**	**Mean ± SD**	**Mean ± SD**	**Mean ± SD**
**SAP** (mmHg)	ISO	118 ± 8.2	133 ± 11.2*	120 ± 21.5	111 ± 12.9	126 ± 10.8*	119 ± 6.2
	KX	117 ± 7.0	144 ± 10.5*	129 ± 19.1*	111 ± 14.3	99 ± 8.1*	113 ± 10.3
	MMF	120 ± 9.1	167 ± 14.1*	170 ± 12.7*	177 ± 15.1*	113 ± 10.0	117 ± 5.2
**DAP** (mmHg)	ISO	87 ± 5.8	97 ± 9.5*	85 ± 19.2	78 ± 12.1*	89 ± 9.1	90 ± 4.6*
	KX	92 ± 9.1	113 ± 8.9*	99 ± 17.3	85 ± 14.2*	76 ± 9.6*	86 ± 5.9
	MMF	89 ± 5.9	124 ± 8.9*	121 ± 9.3*	118 ± 7.6*	86 ± 7.9	91 ± 4.5
**MAP** (mmHg)	ISO	97 ± 6.5	109 ± 10.0*	96 ± 19.9	89 ± 12.3*	101 ± 9.6	100 ± 5.0*
	KX	100 ± 8.0	123 ± 8.9*	109 ± 17.7*	93 ± 13.9*	84 ± 8.5*	95 ± 6.5
	MMF	99 ± 6.9	138 ± 10.6*	137 ± 10.1*	138 ± 9.9*	95 ± 8.5	100 ± 4.6
**PP** (mmHg)	ISO	31 ± 3.8	36 ± 3.5*	35 ± 3.4*	33 ± 2.9*	37 ± 3.7*	30 ± 2.7
	KX	25 ± 5.9	31 ± 6.7*	30 ± 5.9*	26 ± 6.8	23 ± 7.0*	27 ± 8.7
	MMF	31 ± 3.8	43 ± 5.7*	50 ± 5.9*	59 ± 8.5*	27 ± 3.1*	26 ± 2.4*
**HR** (bpm)	ISO	293 ± 18.1	381 ± 35.1*	369 ± 22.0*	404 ± 24.5*	445 ± 20.0*	300 ± 14.1*
	KX	281 ± 22.7	371 ± 33.9*	271 ± 24.0	255 ± 25.7*	289 ± 31.3	316 ± 17.1*
	MMF	293 ± 32.9	303 ± 30.2	229 ± 20.9*	209 ± 24.0*	255 ± 28.6*	286 ± 22.5
**BT** (°C)	ISO	37.34 ± 0.17	37.38 ± 0.23	37.33 ± 0.26	36.56 ± 0.55*	36.39 ± 0.71*	37.34 ± 0.16
	KX	37.34 ± 0.26	37.27 ± 0.25	37.22 ± 0.22	36.78 ± 0.41*	36.95 ± 0.53	37.55 ± 0.45
	MMF	37.49 ± 0.41	37.43 ± 0.40	37.30 ± 0.51	36.90 ± 0.86*	36.54 ± 0.93*	37.25 ± 0.43

No. of animals per treatment: ISO n = 10, KX n = 10, MMF n = 9.

*statistically significant difference (p value ≤ 0.05) based on the ANOVA for the comparison of anaesthetic intervals versus baseline.

**Table 2:** P values based on the ANCOVA

**Parameter**	**Treatment**	**Induction time**		**Non-surgical tolerance**		**Surgical tolerance**		**Wake-up period**		**Recovery**	
		**Mean difference**	**P value**	**Mean difference**	**P value**	**Mean difference**	**P value**	**Mean difference**	**P value**	**Mean difference**	**P value**
**SAP** (mmHg)	ISO vs. KX	-13.18	< 0.01*	-9.15	0.15	-0.58	0.88	26.46	< 0.01*	6.33	0.07
	ISO vs. MMF	-33.07	< 0.01*	-45.79	< 0.01*	-63.08	< 0.01*	15.41	< 0.01*	3.54	0.03*
	KX vs. MMF	-19.88	< 0.01*	-36.64	< 0.01*	-62.50	< 0.01*	-11.05	0.03*	-2.80	0.38
**DAP** (mmHg)	ISO vs. KX	-13.57	< 0.01*	-7.27	0.27	-1.72	0.70	16.39	< 0.01*	5.96	0.01*
	ISO vs. MMF	-27.08	< 0.01*	-33.41	< 0.01*	-38.62	< 0.01*	4.83	0.21	-0.95	0.52
	KX vs. MMF	-13.50	< 0.01*	-26.14	< 0.01*	-36.90	< 0.01*	-11.56	< 0.01*	-6.92	< 0.01*
**MAP** (mmHg)	ISO vs. KX	-13.12	< 0.01*	-7.33	0.25	-0.90	0.83	19.57	< 0.01*	6.14	0.02*
	ISO vs. MMF	-29.09	< 0.01*	-37.48	< 0.01*	-46.74	< 0.01*	8.32	0.05*	0.54	0.71
	KX vs. MMF	-15.98	< 0.01*	-30.16	< 0.01*	-45.84	< 0.01*	-11.25	< 0.01*	-5.60	0.03*
**PP** (mmHg)	ISO vs. KX	-0.76	0.59	2.10	0.26	2.68	0.15	8.41	< 0.01*	-0.44	0.80
	ISO vs. MMF	-6.41	< 0.01*	-13.95	< 0.01*	-25.42	< 0.01*	11.09	< 0.01*	4.55	< 0.01*
	KX vs. MMF	-5.65	< 0.01*	-16.05	< 0.01*	-28.10	< 0.01*	2.69	0.20	4.99	0.02*
**HR** (bpm)	ISO vs. KX	14.62	0.36	93.14	< 0.01*	144.75	< 0.01*	148.62	< 0.01*	-20.86	< 0.01*
	ISO vs. MMF	85.80	< 0.01*	139.93	< 0.01*	195.12	< 0.01*	190.22	< 0.01*	13.41	0.09
	KX vs. MMF	71.17	< 0.01*	46.79	< 0.01*	50.37	< 0.01*	41.60	< 0.01*	34.27	< 0.01*
**BT** (°C)	ISO vs. KX	0.11	0.11	0.10	0.26	-0.22	0.34	-0.56	0.08	-0.21	0.18
	ISO vs. MMF	0.09	0.26	0.16	0.20	-0.19	0.52	-0.04	0.92	0.19	0.17
	KX vs. MMF	-0.01	0.87	0.06	0.67	0.03	0.90	0.52	0.14	0.39	0.04*

*statistically significant difference (p value ≤ 0.05 in bold print) based on the ANCOVA with baseline as covariate for the comparison of anaesthetic treatments.

**Figure 1:**
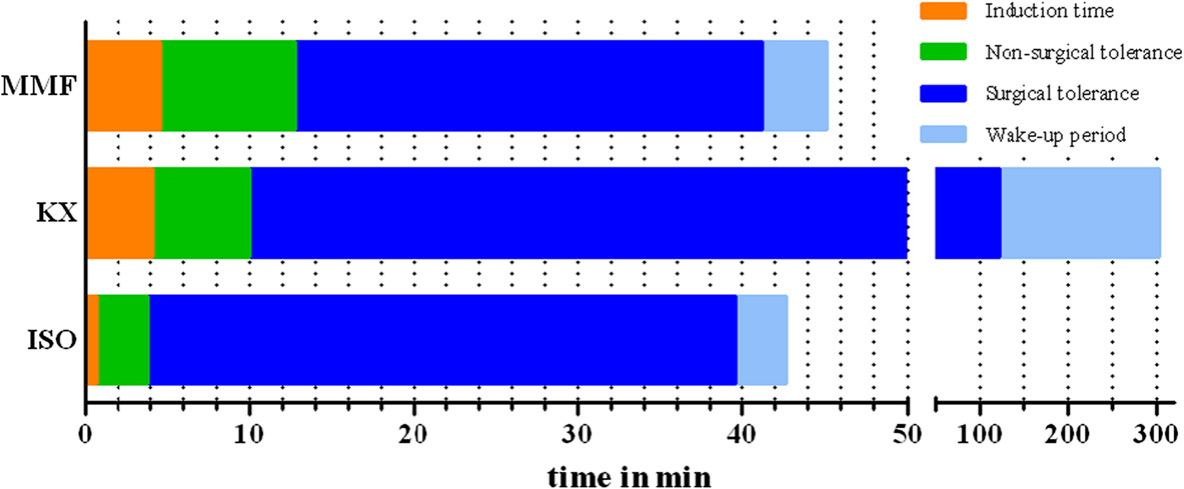
**Mean duration of ISO, KX and MMF anaesthesia.** Anaesthesias were divided in different anaesthetic stages. Induction time (time from application to loss of righting reflex), time of non-surgical tolerance (time from loss of righting reflex until loss of all measured reflexes), time of surgical tolerance (time from loss of all measured reflexes till regain of one reflex), wake-up period (time from regaining one reflex till regain of righting reflex). After 40 minutes ISO delivery was terminated and MMF was reversed with AFN.

Figure 1 shows the mean durations of the anaesthetic stages for ISO, KX and MMF anaesthesia. The durations of induction time (40 sec) and time of non-surgical tolerance (3 min) in ISO were shorter compared to those using KX (4 min, 6 min) and MMF (5 min, 8 min). Surgical tolerance using ISO and MMF lasted for 40 minutes after induction, since ISO delivery was then stopped and MMF was reversed with its antagonists at this point in time. KX anaesthesia was not reversed and the duration of surgical tolerance (110 min) was much longer compared to those of ISO (36 min) or MMF (28 min). The duration of the wake-up period in ISO (3 min) and MMF (4 min) were comparable and much shorter than the wake-up period of KX (182 min).

### ISO

During induction time SAP, DAP and MAP increased significantly to 133, 97, and 109 mmHg, respectively. PP increased significantly to 36 mmHg, HR increased significantly to 381 bpm and BT remained stable at 37.4°C. PP (35 mmHg), HR (369 bpm) and BT (37.3°C) showed only a slight reduction from induction to the time of non-surgical tolerance but remained significantly increased. SAP (120 mmHg), DAP (85 mmHg) and MAP (96 mmHg) decreased almost to their baseline values and showed no significant differences compared to their baseline values. During surgical tolerance PP remained significantly increased with 33 mmHg. SAP decreased to 111 mmHg, DAP and MAP decreased significantly to 78 and 89 mmHg, respectively. HR increased significantly to 404 bpm and BT decreased significantly to 36.6°C. BP values (SAP 126 mmHg, DAP 89 mmHg, MAP 101 mmHg, PP 37 mmHg) started to increase during the wake-up period. HR reached a peak of 445 bpm and BT decreased significantly further to 36.4°C. During recovery time, HR partially normalized to 300 bpm but was still significantly increased compared to baseline and BT returned to the baseline value (37.3°C). BP returned almost to baseline values (SAP 119 mmHg, DAP 90 mmHg, MAP 100 mmHg, PP 30 mmHg) as well but with still significant increases in DAP and MAP compared to baseline.

### KX

BP values (SAP 144 mmHg, DAP 113 mmHg, MAP 123 mmHg, PP 31 mmHg) and HR (371 bpm) increased significantly during the induction time, whereas BT (37.3°C) remained stable. From induction to time of non-surgical tolerance a reduction was observed in all parameters. PP (30 mmHg) was still significantly increased and BT (37.2°C) was not significantly affected. BP values (SAP 129 mmHg, DAP 99 mmHg, MAP 109 mmHg) and HR (271 bpm) came close to baseline values but SAP and MAP showed still a significant increase. From non-surgical to surgical tolerance all parameters showed a decrease and, except PP, reached levels lower than their baseline values (SAP 111 mmHg, DAP 85 mmHg, MAP 93 mmHg, PP 26 mmHg, HR 255 bpm, BT 36.8°C). The difference was significant for DAP, MAP, HR and BT. A significant decrease still could be observed during wake-up period (SAP 99 mmHg, DAP 76 mmHg, MAP 84 mmHg, PP 23 mmHg), except in HR (289 bpm) and BT (37.0°C) which showed values close to their baseline. BP values increased (SAP 113 mmHg, DAP 86 mmHg, MAP 95 mmHg, PP 27 mmHg) once the animals had righted themselves and values returned almost to baseline. HR significantly increased to 316 bpm and BT continued to increase to a final value of 37.6°C.

### MMF

Significant increases in BP values (SAP 167 mmHg, DAP 124 mmHg, MAP 138 mmHg, PP 43 mmHg) were observed during the induction time. HR (303 bpm) and BT (37.4°C) did not change significantly from their baseline values. PP (50 mmHg) and SAP (170 mmHg) continued to increase significantly during the time of non-surgical tolerance and MAP (137 mmHg) and DAP (121 mmHg) showed no great alteration compared to induction time but remained significantly increased. A decrease in HR (from 303 to 229 bpm) was observed from induction time to time of non-surgical tolerance and BT was not significantly affected. During time of surgical tolerance PP reached a maximum of 59 mmHg with a SAP of 177 mmHg, a DAP of 118 mmHg and a MAP of 138 mmHg. HR and BT significantly decreased to 209 bpm and 36.9°C, respectively. During the wake-up period initiated by the administration of the three antagonists, BP parameters (SAP 113 mmHg, DAP 86 mmHg, MAP 95 mmHg, PP 27 mmHg) decreased to levels lower than baseline values, but this change from baseline was only significant for PP. HR (255 bpm) remained significantly decreased. BT further decreased to 36.5°C during wake-up period. BP values (SAP 117 mmHg, DAP 91 mmHg, MAP 100 mmHg), HR (286 bpm) and BT (37.3°C) returned back to baseline values during the time of recovery, except PP (26 mmHg), where a significant decrease still could be observed.

**Figure 2:**
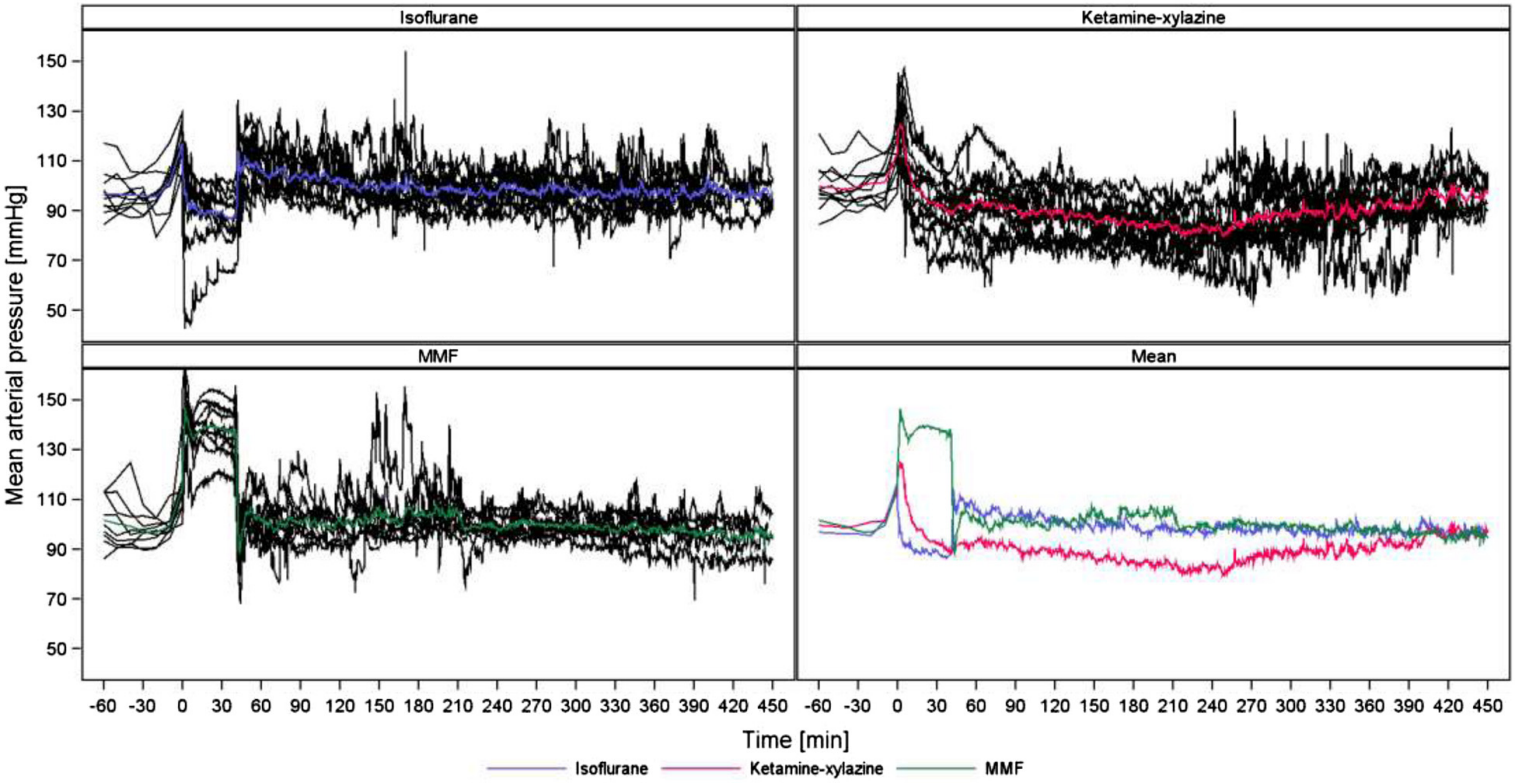
**Mean arterial blood pressure (mmHg).** Individual and mean time courses per treatment group.

**Figure 3:**
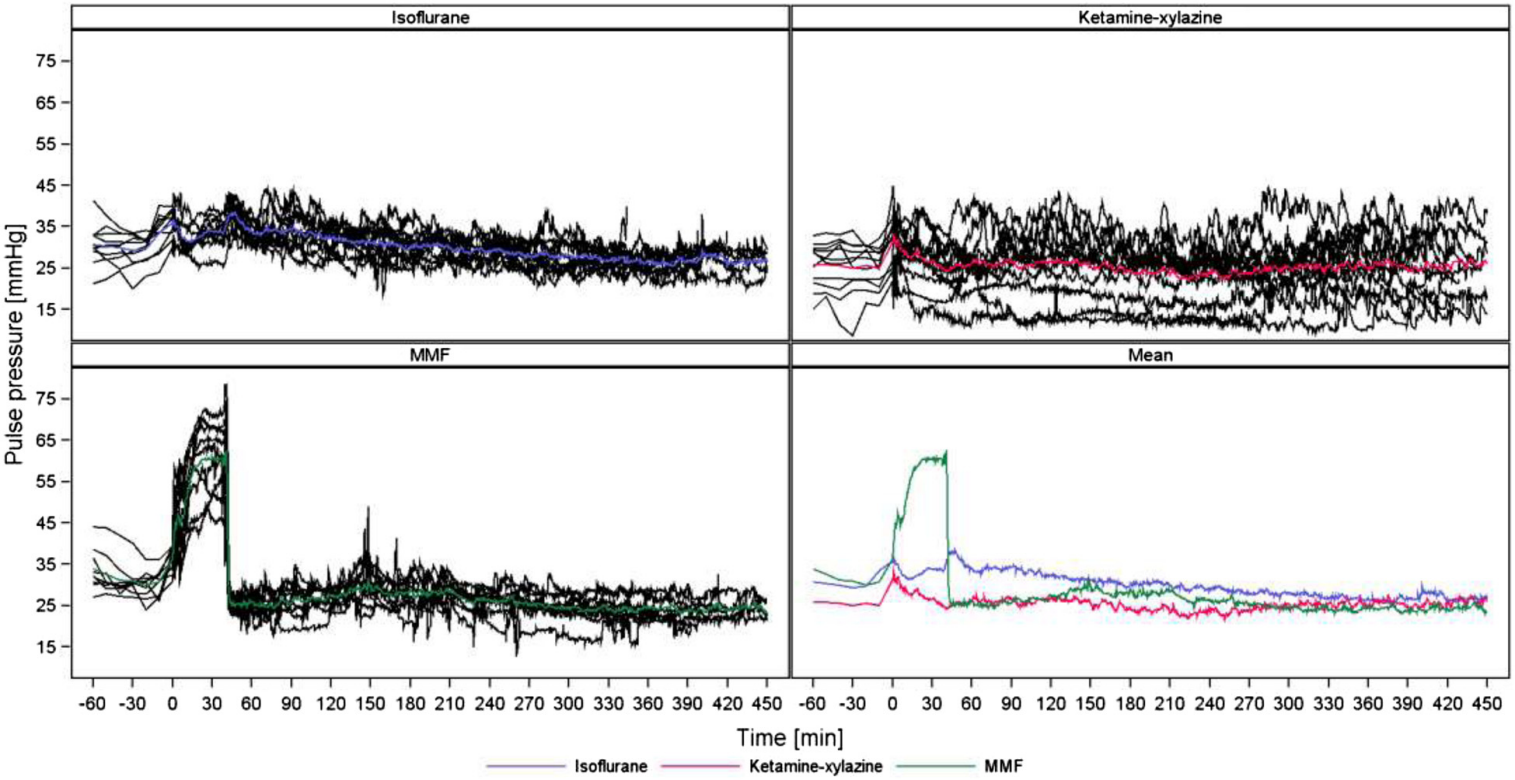
**Pulse pressure (mmHg).** Individual and mean time courses per treatment group.

**Figure 4:**
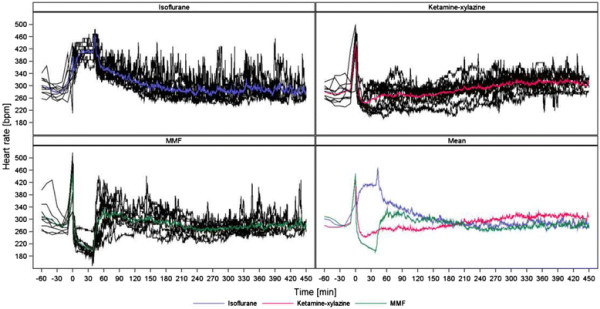
**Heart rate (bpm).** Individual and mean time courses per treatment group.

**Figure 5:**
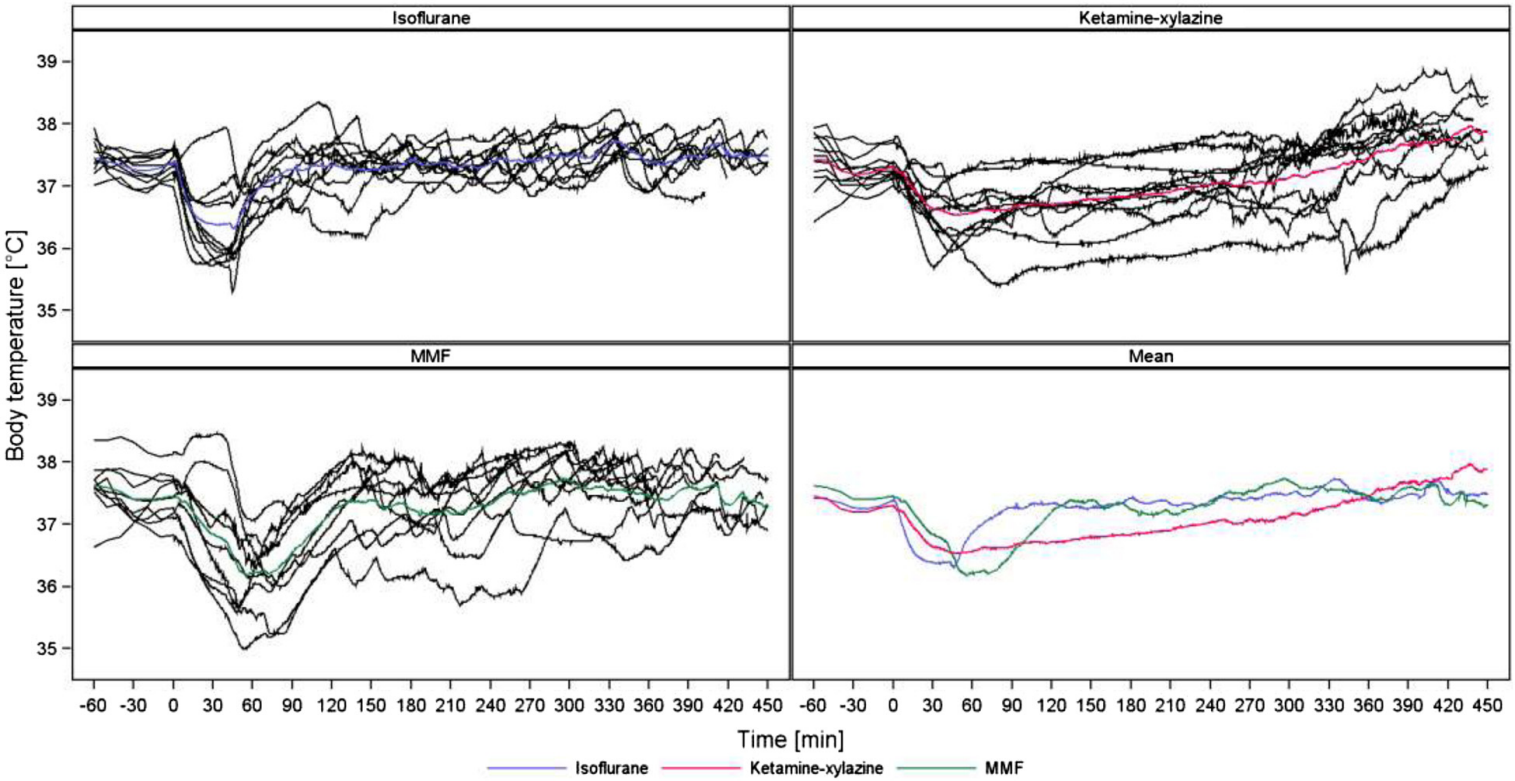
**Core body temperature (°C).** Individual and mean time courses per treatment group.

As demonstrated in Figures 2, 3, 4 and 5 (MAP, PP, HR, BT) the measured parameters showed differences among the anaesthetic treatments. P-values for comparisons of the three treatments with each other are presented in Table 2. Most prominent differences in haemodynamic parameters were observed during anaesthesia if MMF was compared either with ISO or KX. ISO compared with KX showed significant differences in BP values only during induction time, wake-up period and recovery. Significant differences in HR were observed almost in all anaesthetic stages comparing ISO, KX and MMF among each other. On the other hand, BT did not differ significantly between the anaesthesias during the different phases, except during recovery if comparing KX with MMF.

## Discussion

The present study was designed to determine the effect of three anaesthetic regimes (ISO, KX, MMF) in adult, male Wistar rats on cardiovascular parameters and BT. The novel use of telemetric data assessment for these parameters provided accurate and continuous measurements. To our knowledge, such detailed cardiovascular data for ISO, KX and MMF in the rat have never been reported.

ISO, KX and MMF showed significantly different effects on cardiovascular parameters and BT. We evaluated continuously the impact of the anaesthesias and compared them to baseline values assessed in the same rats. It was important to measure baseline values in each rat used in this study under low stress conditions directly before anaesthetic measurement, because baseline values found in the literature show a great variability. In our study mean HR at baseline ranged from 281 to 293 bpm indicating that the rats were well adapted to the experimental environment, because these values are at the low end of those found in literature (250–500 bpm) [1],[3],[20]. Given the comparable baseline data between the treatment groups, we can attribute subsequent changes observed to the three different anaesthesia regimes tested.

### Route of administration

The route of administration is an important consideration. The most convenient and less stressful method to induce anaesthesia in rats is to use an inhalant, like ISO, with an anaesthetic chamber for induction [3],[21]. We used the intramuscular route in the other two treatment groups because intraperitoneal injection may cause more stress to the animals and there is a risk of up to 30% for misdirected injections into the gastrointestinal tract or the urinary bladder [20],[38]–[40]. Furthermore even a small failure rate of i.p. injections may have serious consequences for analysis and data interpretation [41]. For MMF the intramuscular application route worked well but with KX we observed defensive movements of the rats during injection and in three animals we noticed local tissue necrosis at the injection site one week after anaesthesia. The volumes being injected were comparable for MMF (0.65 ml•kg^−1^) and KX (2 × 0.625 ml•kg^−1^) when considering that the volume of KX was divided in half and being injected in the femoral muscles of both hind legs. Therefore, we propose that the acidic formulation of ketamine was responsible for this severe tissue necrosis and not the amount of injected anaesthetics. Other studies have reported local tissue necrosis or self-mutilation after an intramuscular injection of ketamine or combinations including it [42]–[46]. Due to these facts the application route for KX and the use of a less acidic formulation of ketamine should be considered for further investigations.

### Duration of anaesthesia

The planned duration of anaesthesia was 40 minutes. Many types of surgical procedures can be performed during this timeframe and other comparative studies selected similar durations for anaesthesia [24],[25],[42]–[45]. As shown in Figure 1, ISO and MMF are best suited for this intention, whereas the mean duration of KX anaesthesia lasted 301 minutes. MMF was quickly reversed with a subcutaneous injection of AFN and anaesthesia with ISO was reversed quickly after stopping ISO delivery. The possibility of quickly terminating anaesthesia with ISO and MMF and the resultant short wake-up period provide distinct advantages compared to KX anaesthesia for some procedures. The xylazine-dependent portion of the KX anaesthesia could have been antagonized. In two studies the α_2_-adrenoceptor antagonist atipamezole was used after KX and ketamine-medetomidine anaesthesia in mice, to reverse the effects of the α_2_-adrenoceptor agonists, xylazine and medetomidine, respectively. However, it was reported that mice need more time for recovery after early reversal, compared to a later partial antagonisation after 40 minutes [47],[48]. Therefore, if using atipamezole for reversal of KX anaesthesia in rats, it has to be considered which is the most suitable moment for the administration in this species. In regard to the long duration of wake-up and recovery period a reversal with atipamezole is recommendable, although ketamine induced effects (increased muscle tone, increased catecholamine levels) could still be observed if administering atipamezole- too early [21],[49].

### Effects on blood pressure

BP was affected variably by the three anaesthetic regimes. Due to handling and anaesthetic administration stress (i.m. injection or exposure to an irritant gas) SAP, DAP, MAP and PP increased transiently during administration in all of the three regimes. In contrast to ISO and KX, the BP values with MMF were substantially higher than with the other two up to and including the time of surgical tolerance. BP during ISO and KX tended to decrease compared to their baseline values. BP values with ISO were lowest during the time of surgical tolerance with a MAP of 89 mmHg. Vasodilative properties of ISO can lead to serious hypotension [17],[20], but our lowest measured values never indicated a marked hypotension. The administration of xylazine, an α_2_-adrenoceptor agonist, results in an initial increase in BP but changes to a long-lasting hypotension. To produce a general anaesthesia and to compensate for the negative effects of xylazine on BP it is recommended to combine xylazine with the dissociative anaesthetic agent ketamine because of its stimulating effects on the central nervous system [20]. Although the combination of KX should therefore not lead to a marked hypotension, BP continuously decreased over a long time to mild hypotensive values almost until the rats had regained their righting reflex. These findings are in accordance with another study, which showed the most prominent hypotension after 150 minutes of KX anaesthesia in rats [50]. In contrast, MMF had a completely different effect on BP. MMF has been reported to provide stable haemodynamic conditions, but a marked decrease of BP after its reversal [20],[24],[25]. Values were often only presented as MAP, so the real extent of the increase of SAP, DAP and PP remained unclear. Previous studies with MMF anaesthesia found a notable increase of MAP in rats, but not in rabbits, mongolian gerbils and chinchillas [22],[24],[25],[32],[51]–[54]. Another study evaluated MMF in the syrian golden hamster and based on that data is was suspected that different ages of the hamsters could have had a different effect on BP [55]. In mice, a decrease of BP parameters to lower values compared to a KX anaesthesia was reported [56]. Another study described an increase of BP in dogs (beagles) during medetomidin-midazolam-butorphanol and medetomidin-midazolam-buprenorphine anaesthesia and a significant decrease after antagonization with atipamezole [57]. Based on these various effects of MMF on cardiovascular parameters, there is the necessity of further studies, which evaluate the influence of this anaesthetic combination in different species. MMF in the present study produced a significant increase in all BP parameters. With such hypertensive values it is not recommended to perform surgical procedures on large blood vessels, due to a greater risk of bleedings. Retrospectively, we noticed that the implantation of our telemetry device performed under MMF anaesthesia lead to a higher bleeding risk during surgery than when using ISO anaesthesia. The reason for choosing MMF for the implantation surgery for our study was the better analgesic properties of MMF compared to ISO. As described in literature, we also observed a decrease of BP after injection of the antagonists. Although these values were lower than their baseline values we do not consider them to indicate a marked hypotension, especially since they lasted for less than two minutes. However, one published study stated that the antagonization of MMF during an anaesthetic emergency could result in a haemodynamically critical and life-threatening state because of the decreased BP [24]. In a further study, the reversal of MMF after an induced hemorrhagic shock in rats did not lead to life-threatening haemodynamic conditions [58].

### Methods for blood pressure measurement

BP values reported in this study are different from values reported in other studies. A likely explanation is the method of measuring BP parameters. The direct measurement of BP in the abdominal aorta as used in the present study provides more precise data than a tail-cuff system or an exteriorized catheter in the *A. carotis* or *A. femoralis*[37],[59]. Exteriorized catheters, despite routine flushing, will eventually clot and lose their ability to transmit high frequency pressure signals. Their utility is thereby limited and cannot be maintained for as long as for animals implanted with a telemetry device. Furthermore, a complete recovery from a catheter implantation surgery under normal group-housing conditions is not possible. Thus for the present study, using a cross-over experimental design with adequate washout periods between the anaesthesias, the implantable telemetry-based system was clearly advantageous.

### Effects on heart rate

Differences in HR were present in all stages of anaesthesia among the groups receiving ISO, MMF and KX. Low BP values with ISO due to its vasodilative properties may have led to the observed increase in HR through baroreceptor activation. The highest HR being observed during the wake-up period has not been observed previously. One might expect an increase in BP with the loss of vasodilative effects of ISO during the wake-up period with a decrease in HR. However, we noticed that BP as well as HR increased after terminating ISO delivery, which may reflect a recovery of sympathetic tone. The ISO-induced increase of HR should be taken into account in the design of studies assessing drug-induced effects. When using MMF or KX, stress associated with the injection caused an initial increase of HR followed by a rapid decrease lower than baseline. With KX, the lowest HR was observed after 30 minutes followed by a continuous, slow increase of HR almost until the end of the measurement. One study described similar findings of alterations in HR during KX anaesthesia in rats and it was suggested that the cardiovascular depressant effect of xylazine overrides the increased sympathetic and reduced vagal tone and the adverse effect of baroreceptor reflex attributed to ketamine [50]. HR during MMF rapidly decreased after injection and continued to decrease slightly until it was antagonized. It is known that the α_2_-adrenoceptor agonist medetomidine and the opioid fentanyl can lead to dose-dependent bradycardia [3],[20] and the dosage needed for MMF anaesthesia were associated with bradycardia. Other studies were in accordance with this finding as the authors noted a decrease of HR in rats, rabbits and chinchillas during MMF anaesthesia until antagonization [24],[25],[32],[51],[52].

### Effects on body temperature

It is known that anaesthetics alter thermoregulation [3],[20]. Hypothermia impedes metabolism of anaesthetics, prolongs the wake-up period and is a frequent cause of post-anaesthetic deaths [3],[16]. Small animals like rodents rapidly lose body heat because of their relatively large surface to volume ratio [3],[20]. Although there was only a significant difference of BT between KX and MMF during recovery, all anaesthetic regimes showed a notable decrease in BT after induction of anaesthesia. MMF and ISO showed their lowest values during the wake-up period, whereas BT in KX had its lowest value during time of surgical tolerance (about 40 minutes after injection). During the wake-up period a significant decrease of BT could no longer be observed with KX. We suggest that the normalization of BT in KX during anaesthesia was caused by an increased muscle tone due to the cataleptic property of ketamine and mainly by a long lasting wake-up period where animals slowly regain thermoregulatory control. The wake-up period durations of MMF and ISO were too short to produce a significant impact on BT because of regaining consciousness and thermoregulatory control, therefore BT showed normalization only during the time of recovery. In contrast to our findings, another study, evaluating parameters during KX anaesthesia in rats, showed the lowest value of BT at 150 minutes after injection. In that study it is not mentioned whether heat was provided during anaesthesia [48]. Compared to a study which evaluated the effects of KX, MMF and ISO on BT in mice and observed the greatest decrease of BT during ISO anaesthesia, our measurements showed no significant differences in the decrease of BT during anaesthesia. In that study heat-supply is not only recommended during anaesthesia, but also during the preanaesthetic period [60]. It seems that proving warmth, as done in the present study throughout the procedure, cannot completely prevent a decrease of BT during anaesthesia, but it may effectively reduce the extent of heat-loss.

## Conclusion

Our study compared the haemodynamic effects of ISO, KX and MMF anaesthesia in chronically instrumented rats using telemetric data collection.

ISO caused a mild hypotension and a significant increase of HR during anaesthesia. But with ISO, the greatest increase of HR was observed in the wake-up period. BP as well as HR decreased significantly using KX and while HR returned to baseline during wake-up period, BP continued to decrease until the animals regained consciousness. Marked increases of BP values and a significant decrease of HR were observed with MMF. Although these effects were completely reversed with an injection of AFN, in MMF the increased bleeding risk when manipulating large blood vessels should be kept in mind.

ISO, KX and MMF all have advantages and disadvantages, but which anaesthesia is most suitable for an experimental investigation depends on the aim of the study. Without partial antagonization with atipamezole we would not recommend KX for experimental procedures requiring a quick recovery and since it caused tissue necrosis at the injection site, when administered i.m.

## Abbreviations

AFN: Atipamezole-flumazenil-naloxone

BP: Blood pressure

bpm: Beats per minute

BT: Body temperature

DAP: Diastolic arterial pressure

DSI™: Data Science International™

Fig.: Figure

HR: Heart rate

i.m.: Intramuscular

ISO: Isoflurane

KX: Ketamine-xylazine

MAP: Mean arterial pressure

min: Minute

MMF: Medetomidine-midazolam-fentanyl

PP: Pulse pressure

SAP: Systolic arterial pressure

s.c.: Subcutaneous

sec: Second

SD: Standard deviation

## Competing interests

None of the authors of this paper has a financial or personal relationship with other people or organizations that could inappropriately influence or bias the content of the paper. The authors declare that they have no competing interests.

## Authors’ contributions

MA developed the experimental design of the study, performed the transmitter implantation surgery and all anaesthesias, prepared and interpreted data, created tables and figures, drafted and finalized the manuscript. JH initiated the study, developed the experimental design, analyzed and interpreted data and revised the manuscript. ST was involved in developing the study design, discussed the result with MA and JH and revised the manuscript. MM developed the experimental design of the study, analyzed and interpreted data and revised the manuscript and supervised the study. BG is the supervisor of the experimental group in which the studies were conducted, actively participated in the interpretation of the data as well as in the editing of the manuscript. All authors read and approved the final manuscript.
